# Morphological and molecular characteristics of spheroid formation in HT-29 and Caco-2 colorectal cancer cell lines

**DOI:** 10.1186/s12935-021-01898-9

**Published:** 2021-04-13

**Authors:** Elmira Gheytanchi, Marzieh Naseri, Feridoun Karimi-Busheri, Fatemeh Atyabi, Ensie Sadat Mirsharif, Mahmood Bozorgmehr, Roya Ghods, Zahra Madjd

**Affiliations:** 1 Oncopathology Research Center, Iran University of Medical Sciences, Tehran, Iran; 2 Department of Molecular Medicine, Faculty of Advanced Technologies in Medicine, Iran University of Medical Sciences, Tehran, Iran; 3 Department of Oncology, University of Alberta, Edmonton, Alberta Canada; 4 Nanotechnology Research Centre, Department of Pharmaceutics, Faculty of Pharmacy, Tehran University of Medical Sciences, Tehran, Iran; 5 Immunoregulation Research Center, Shahed University, Tehran, Iran

**Keywords:** Colorectal cancer (CRC), Cancer stem cells (CSCs), Sphere formation, Epithelial-to-mesenchymal transition (EMT), Drug resistance

## Abstract

**Background:**

Relapse and metastasis in colorectal cancer (CRC) are often attributed to cancer stem-like cells (CSCs), as small sub-population of tumor cells with ability of drug resistance. Accordingly, development of appropriate models to investigate CSCs biology and establishment of effective therapeutic strategies is warranted. Hence, we aimed to assess the capability of two widely used and important colorectal cancer cell lines, HT-29 and Caco-2, in generating spheroids and their detailed morphological and molecular characteristics.

**Methods:**

CRC spheroids were developed using hanging drop and forced floating in serum-free and non-attachment conditions and their morphological features were evaluated by scanning electron microscopy (SEM). Then, the potential of CSCs enrichment in spheroids was compared to their adherent counterparts by analysis of serial sphere formation capacity, real-time PCR of key stemness genes (*KLF4*, *OCT4*, *SOX2*, *NANOG*, *C-MYC*) and the expression of potential CRC-CSCs surface markers (CD166, CD44, and CD133) by flow cytometry. Finally, the expression level of some EMT-related (*Vimentin*, *SNAIL1*, *TWIST1*, *N-cadherin*, *E-cadherin*, *ZEB1*) and multi-drug resistant (*ABCB1*, *ABCC1*, *ABCG2*) genes was evaluated.

**Results:**

Although with different morphological features, both cell lines were formed CSCs-enriched spheroids, indicated by ability to serial sphere formation, significant up-regulation of stemness genes, *SOX2*, *C-MYC, NANOG* and *OCT4* in HT-29 and *SOX2*, *C-MYC* and *KLF4* in Caco-2 spheroids (*p-value* < *0.05*) and increased expression of CRC-CSC markers compared to parental cells (*p-value* < *0.05*). Additionally, HT-29 spheroids exhibited a significant higher expression of both *ABCB1* and *ABCG2* (*p-value* = *0.02*). The significant up-regulation of promoting EMT genes, *ZEB1*, *TWIST1*, *E-cadherin* and *SNAIL1* in HT-29 spheroids (*p-value* = *0.03*), *SNAIL1* and *Vimentin* in Caco-2 spheroids (*p-value* < *0.05*) and *N-cadherin* down-regulation in both spheroids were observed.

**Conclusion:**

Enrichment of CSC-related features in HT-29 and Caco-2 (for the first time without applying special scaffold/biochemical) spheroids, suggests spheroid culture as robust, reproducible, simple and cost-effective model to imitate the complexity of in vivo tumors including self-renewal, drug resistance and invasion for in vitro research of CRC-CSCs.

## Background

Colorectal cancer (CRC) is the third most common cancer and the cause of cancer related-death in both men and women worldwide [1]. Although the effective therapeutic interventions including surgery, chemo- and radiotherapy have improved the survival of patients with CRC, recurrence and metastasis are the main causes of CRC-related death [2]. There is accumulating evidence that most solid tumors including CRC originate from tumor initiating or cancer stem cells (CSCs) [3–5]. CSCs are referred to as a sub-population of tumor cells with stem cell-like properties including self-renewal and multi-lineage differentiation capacity which are resistant to conventional therapies [6–9]. In fact, the emerging evidence has shown that the deregulation of signaling pathways involved in self-renewal of normal stem cells leads to uncontrolled development of tumor cells along with functional and proliferative tumor heterogeneity [10].

Most of the challenges in cancer treatment such as treatment failure, tumor aggressiveness, relapse, metastasis and poor prognosis are related to CSCs characteristic, most of which are attributed to epithelial to mesenchymal transition (EMT) potential, stemness signaling pathways regulating pluripotency, as well as the high expression of ATP-binding cassette (ABC) transporter genes in this subpopulation [11–18]. Hence, the complete regression of tumors requires identifying biological features of CSCs in in vitro and in vivo CSC models that may help to explore new approaches to target this subpopulation. There are several strategies of CSCs targeting including self-renewal prevention, altering drug permeability and stimulation of their differentiation [19–21]. Moreover, signal transduction pathways and microenvironmental signals in CSCs are considered the main therapeutic aspects linked to stem-cell biology that can be targeted [13, 22, 23].

Since it is difficult to fully identify the site of CSCs accumulation and monitor their distinct biological features, the discovery and development of novel techniques for the isolation and evaluation of CSCs properties can play a significant role in the clinical diagnosis and treatment of cancers. Different approaches have been applied to isolate, characterize, purify, and enrichment of CSCs from various cancers [7, 24]. Immunogenic characteristics of CSCs, particularly as related to their specific cell surface markers such as CD133, ALDH1 and CD44, are the well-known methods which apply for CSCs isolation and characterization in the early diagnosis and targeted therapy of cancer [25], while methods such as sphere formation, clonogenic growth and drug resistance assays lead to functional isolation of CSCs [25].Thus, the development of feasible and applicable models for CSCs enrichment and assessment of its characteristics are warranted. Cell-based assays including in vitro cell culture, multicellular spheroids and organoid culture are valuable tools in obtaining information regarding cellular and molecular mechanisms involved in CSCs biology, and exploring new therapeutic strategies for targeting this sub-population [24, 26].

It is well accepted that micro tumor structures formed as spheroids present the main characteristics of in vivo tumors such as cellular heterogeneity, hypoxia and pH rate, exposure to nutrients and metabolites, cell–cell cohesion and interaction, physical and chemical stresses, and gene expression profiles [27]. Therefore, spheroids with their particular architecture and biology can serve as reliable cancer models in different areas of cancer research, including tumor microenvironment modeling and matrix remodeling as well as migration and invasion, drug discovery and screening, immune interactions and angiogenesis, etc. [26, 28, 29]. There are different systems of spheroid formation, including suspension culture, non-adherent surface, hanging drop and microfluidic methods. Spheroids culture as serum free and non-adherent condition is the most frequent and powerful technique to obtain CSC-like properties and expand CSCs. Moreover, the presence of mathematically testable approaches based on destruction of spheroids structure decreases underestimated results in clinical application and guarantees reproducibility [30, 31]. CRC cell lines have derived from different CRC subtypes and show different phonotypic and molecular properties in in vivo and in vitro studies [32, 33]. For example, despite similar epithelioid phenotypes of HT-29 and Caco-2 cells, HT-29 cells are invasive and metastatic in vivo, but not in vitro, whereas Caco-2 cells are noninvasive [34]. Moreover, the different cell lines can generate spheroids with differences in morphological feature, as well as the capacity of CSCs enrichment and CSC characteristics-related gene expression profiles. To overcome these limitations it is necessary to determine the ideal, most suitable cell line for CSC studies both through evaluation and comparison of their-derived spheroids in terms of stem cell-like characteristics.

Considering the aforementioned, this study aims to gain insight into the biological features of two different types of colonospheres, both derived from two colorectal cancer cell lines including HT-29 and Caco-2 and assess whether these spheres are suitable models for CSC enrichment. Then, we evaluated the CSC-related characteristics of generated spheroids in terms of gene expression profile and morphological features as compared to their parental cells in two-dimensional (2D) culture. Hence, we compared the expression level of genes, as related to the CSCs properties, including genes implicated in stemness, EMT and drug resistance in HT-29 and Caco-2 human colorectal carcinoma (CRC) cell lines grown in spheroids versus 2D cell culture conditions. We applied cost-effective, simple, and time-consuming methods for spheroid formation without using special scaffold or biochemical materials.

## Materials and methods

### Cell lines and culture conditions

HT-29 and Caco-2 human colon adenocarcinoma cell lines were obtained from the Iranian Biological Resource Center (IBRC, Tehran, Iran) and were tested for spheroid formation. Cells were cultured in Dulbecco's modified Eagle's medium (DMEM)/High glucose medium (Gibco, Germany) supplemented with 10% fetal bovine serum (FBS, Gibco, Germany), 100 U/ml penicillin, 100 µg/ml streptomycin antibiotics (Biowest, France), 1% non-essential amino acid (Gibco, Germany) and 2 mM l-glutamine (Gibco, Germany). Cultures were maintained under standard cell culture conditions in 37 °C, 5% CO2 and 95% humidified incubator and passaged in 70–90% confluence.

### Preparation of poly-HEMA coated cell culture dishes

To prepare the 1.2% poly-HEMA (Poly-2-hydroxyethyl methacrylate) solution, 1.2 g poly-HEMA (Sigma, USA) was dissolved in 100 mL 96% ethanol by rotating overnight to dissolve the polymers completely. The solution was centrifuged for 30 min at 400×*g* to remove unsolved particles and then filtered by 0.22 µm filters. The tissue culture dishes were coated with poly-HEMA solution (1.2 mL per each well of six well plate or 2.5 mL per T25 tissue culture flask) under the biosafety laminar flow hood at room temperature for an overnight to evaporate ethanol completely. Finally, the plates were washed with phosphate buffer saline (PBS) and stored at 37 °C incubator for future use.

### Spheroids culture

HT-29 and Caco-2 spheroids were generated by two types of spheroid culture systems; the hanging droplet technique or as free-floating spheroids cultured on poly-HEMA coated dishes. For hanging droplet spheroids, the cells were detached with 0.05% trypsin/EDTA (Gibco, Germany) and after trypsin inactivation, the resulting single cells were washed twice with PBS and pre-warmed serum free media. Dissociated single cells re-suspended as five or ten thousand cells per 25μL of serum free medium (DMEM/F12, Gibco, Germany), which was supplemented with 20 ng/mL epidermal growth factor (EGF, PeproTech, USA), 10 ng/mL of basic fibroblast growth factor (bFGF, PeproTech, USA), 2% B27 supplement (Gibco, Germany), 1% non-essential amino acid, 2 mM l-glutamine and 1% penicillin–streptomycin. About 60 × 25μL cellular drops were dispensed on the inverted lids of the 9 cm petri dishes and the lids carefully set on the dishes, which had been pre-filled with five mL of PBS to assure high humidity and then incubated for 96 h. In the next step, the droplets were washed with 2 mL of media by gentle shaking, and formed spheroids were transferred onto poly-HEMA coated dishes for an additional 6 days. For free-floating spheroids, single-cell suspensions were seeded into poly-HEMA coated dishes at different cell densities (1–5 × 10^5^ cells/mL) in the serum free medium as described above and were cultivated for up to 10 days. The culture media was supplemented with additional 2% B27, bFGF and EGF every other day.

### Secondary sphere formation assay

To examine the ability of colonospheres to form the next spheroid generations, spheroids were harvested and dissociated enzymatically with trypsin/EDTA and mechanically by gently pipetting. The resulting single cells, after counting, were re-plated in serum free spheroid medium at the same densities and culture conditions as mentioned above for three sequential passages (P1-P4).

### Scanning electron microscopy

Spheroids were collected by centrifugation for 1 min at 100 g and supernatant were carefully aspirated. Then, the collected spheroids were washed with PBS and fixed with 2.5% (v/v) glutaraldehyde in PBS for 30 min at room temperature. After fixation, spheroids were washed once with PBS and dehydrated using ethanol series (50, 65, 75, 85, and 100%). The samples were sputter-coated with gold–palladium and examined in scanning electron microscope (SEM, Seron Technology, AIS-2100, Korea).

### Quantitative real-time PCR analysis

The following genes were selected and examined by real-time PCR: stemness genes; *KLF4*, *OCT4*, *SOX2*, *NANOG* and *C-MYC*, EMT genes; *Vimentin*, *SNAIL1*,*TWIST1*, *N-cadherin*, *E-cadherin* and *ZEB1*, ABC transporter genes; *ABCB1*, *ABCC1* and *ABCG2*. Spheroids were harvested a day before structural disintegration (day 10 for HT-29 and day 4 for Caco-2 spheroids). The total RNAs were then extracted from parental and spheroid cells using RNeasy Mini Kit (Qiagen, Germany) according to the manufacturer’s instructions. After measurement of RNA quantity and quality by Nanodrop (ThermoFisher Scientific, USA), cDNA were synthesized with 1 μg of total RNA using cDNA synthesis kit (GeneAll, Korea). Real-time polymerase chain reaction (RT-qPCR) was performed using the SYBR Premix Ex Taq II real-time PCR kit (TaKaRa, Japan) on the Rotor-Gene Q LightCycler (Qiagene, Germany) with the following conditions: 40 two-step amplification cycles of 95 °C for 5 s and 60 °C for 30 s. The relative expression values of target genes were quantified relative to glyceraldehyde-3-phosphate dehydrogenase (*GAPDH*), as the internal reference gene, by using the 2^−ΔCT^ method. Real-time PCR primers are listed in Table 1.

**Table1:** Primers used for quantitative RT-PCR

Genes groups	Gene name	Primer Sequence (5´ → 3´)
Housekeeping gene	*GAPDH*	F-CATGAGAAGTATGACAACAGCCT R-AGTCCTTCCACGATACCAAAGT
Stemness genes	*C-MYC*	F-ACACATCAGCACAACTACG R-CGCCTCTTGACATTCTCC
	*KLF4*	F-CCTCGCCTTACACATGAAGAG R-CATCGGGAAGACAGTGTGAAA
	*SOX2*	F-AATGGGAGGGGTGCAAAAGAGG R-GTGAGTGTGGATGGGATTGGTG
	*NANOG*	F-AGCTACAAACAGGTGAAGAC R-GGTGGTAGGAAGAGTAAAGG
	*OCT4-A*	F-GTGGAGAGCAACTCCGATG R-TGCAGAGCTTTGATGTCCTG
EMT genes	*Vimentin*	F-TCTACGAGGAGGAGATGCGG R-GGTCAAGACGTGCCAGAGAC
	*SNAIL1*	F-CCAGAGTTTACCTTCCAGCA R-GATGAGCATTGGCAGCGA
	*TWIST1*	F-TTCTCGGTCTGGAGGATGGA R-CCACGCCCTGTTTCTTTGAAT
	*N-cadherin*	F-GCCCAAGACAAAGAGACCC R-CTGCTGACTCCTTCACTGAC
	*E-cadherin*	F-CAGGAGTCATCAGTGTGGT R-GGAGGATTATCGTTGGTGTCAG
	*ZEB1*	F-CTTCTCACACTCTGGGTCTTATTC R-CGTTCTTCCGCTTCTCTCTTAC
ABC Transporte	*ABCG2*	F-TTCCACGATATGGATTTACGG R-GTTTCCTGTTGCATTGAGTCC
	*ABCB1*	F-GTTCAGGTGGCTCTGGATAAG R-AGCGATGACGTCAGCATTAC
	*ABCC1*	F-CGCCTTCGCTGAGTTCCT R-TGCGGTGCTGTTGTGGTG

### Flow cytometry

Flow cytometry was used to quantify the percentage of CSC markers expression in HT-29 and Caco-2 spheroid cells compared to parental cells. The parental and spheroid cells from each cell line were dissociated with trypsin/EDTA and were washed with PBS twice. The dissociated cells were counted using Trypan blue exclusion assay, and if cell viability was more than 95%, they were evaluated for CSC markers expression. The following antibodies were used: anti-CD44 (1:30), anti-CD133 (1:300), anti-CD166 (1:90) (all from abcam, USA). All antibodies were incubated with 3 × 10^5^ cells for 30 min at 4 °C. Goat anti-rabbit IgG-FITC (1:100) (Santa Cruz biotechnology, USA) was used as secondary antibody. The percentage of CSC marker positive cells were evaluated using an Atuune NxT flow cytometer (Thermo Fisher Scientific, USA) and data were analyzed using FlowJo VX software.

### Statistical analysis

Data were reported as the mean ± standard deviation for each group from three or four independent experiments. The diameter of the spheroids was measured using Image J software (n = 25) (IJ 1.46r version, NIH, USA). Student’s t-test was used to compare the differences between the control (parental) and spheroid groups using GraphPad Prism version 8.0 for Windows (GraphPad Software, La Jolla, CA, USA, www.graphpad.com). A *p-value* < *0.05* was accepted as a statistically significant difference between groups.

## Results

### HT-29 and Caco-2-derived spheroids exhibit different spheroidization time and morphological characteristics

**Fig. 1:**
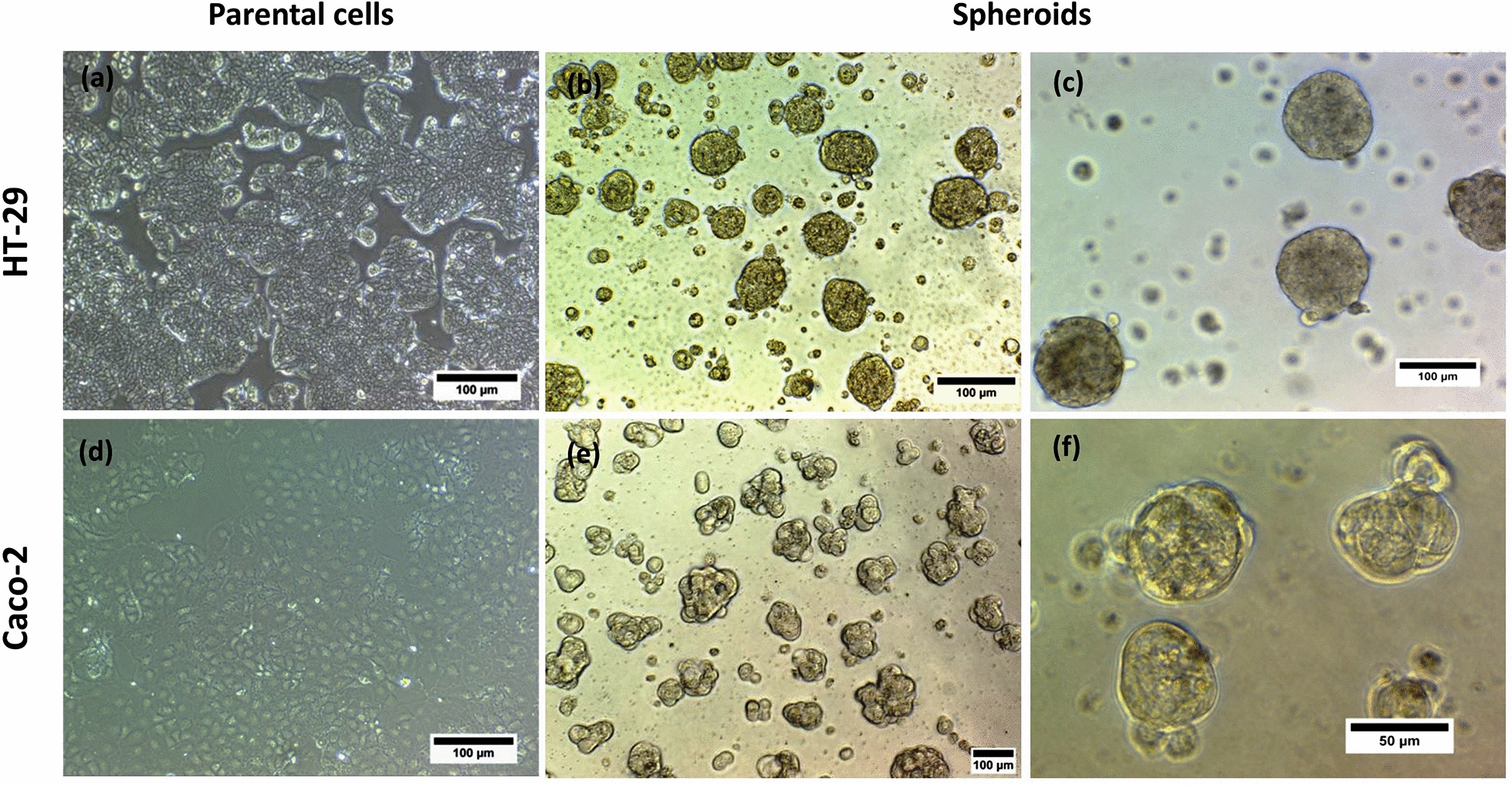
Morphology of HT-29 and Caco-2 parental adherent monolayer cells and their derived spheroids. Representative phase contrast images from cell lines and their derived spheroids. **a** Parental HT-29 cells grew as an adherent monolayer, **b**, **c** HT-29 derived spheroids cultured at nonadherent and serum free condition showed well-round shape and compact morphology. **d** Caco-2 parental cells as monolayer and, **e**, **f** Caco-2 derived spheroids displayed small and round shape morphology

**Fig. 2:**
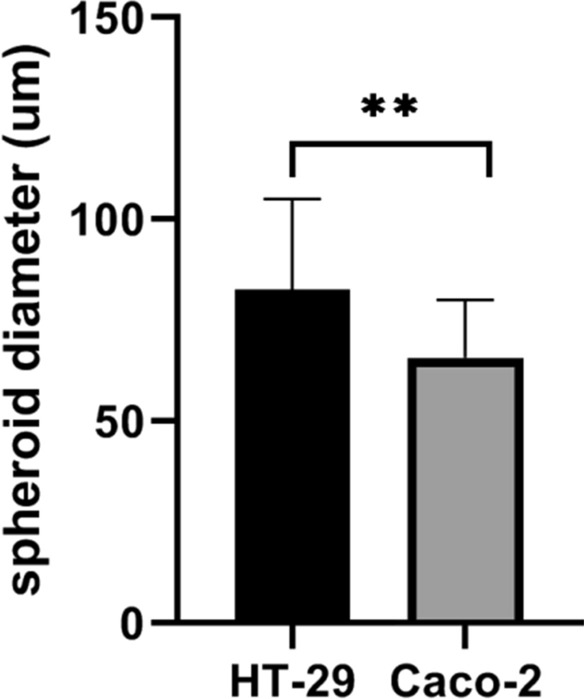
Size comparison of HT-29 and Caco-2 derived spheroids. Caco-2 spheroids showed significantly smaller size in compared to HT-29 spheroids. The average diameter of Caco-2 spheroids was 66.9 ± 14.88 µm compared to 82.52 ± 22.56 µm in HT-29 spheroids. Data are presented as mean ± SD as ** = P < 0.01, (n = 25)

First, we tested whether HT-29 and Caco-2 cell lines (Fig. 1a, d) are able to generate three-dimensional (3D) spheroids. Spheroid formation in non-adherent condition on poly-HEMA coated dishes at different cell densities was applied for generation of spheroids from HT-29 and Caco-2 adherent cells. Both cell lines could form spheroids, while HT-29 spheroids were unstable and the first signs of disintegration of their structure were observed 3 days after culture (data not shown). Hence, we used the hanging drop method as a well-established technique to spheroid formation from HT-29 cells. As observed from Fig. 1, both cell lines formed 3D spheroids in serum free media using the hanging drop method for HT-29 and free floating culture in non-adherent condition for Caco-2. Phase contrast microscope images from spheroids revealed different growth patterns; HT-29 cells formed spheroids with round-type, smooth surface and compact morphology after 96 h incubation as hanging drops and became more compact and dense during 10 days of culture (Fig. 1b, c). By contrast, Caco-2 cells spontaneously started to form spheroids with round-shape structure, and after cultivation for 3 days; they generated hollow spheroids with bubble-like structures (Fig. 1e, f). Furthermore, the size of Caco-2 spheroids was significantly smaller than HT-29 spheroids after 4 days of incubation (*p-value* < *0.01*). The average diameter of Caco-2 spheroids was 66.9 ± 14.88 µm compared to 82.52 ± 22.56 µm in HT-29 spheroids (Fig. 2).

However, Caco-2 cells exhibited shorter spheroidization time; 2 days following plating compared to 4 days in HT-29, whereas HT-29 spheroids were more stable and maintained their compact spheroid architecture even up to 12 days of cultivation. In contrast, Caco-2 spheroids remained stable for 5 days. HT-29 spheroids showed greater increase in diameters over time than Caco-2 spheroids. Whereas, the spheroids derived from Caco-2 cells were slowly growing in size and displayed more stable diameter and less variability in size.

**Fig. 3:**
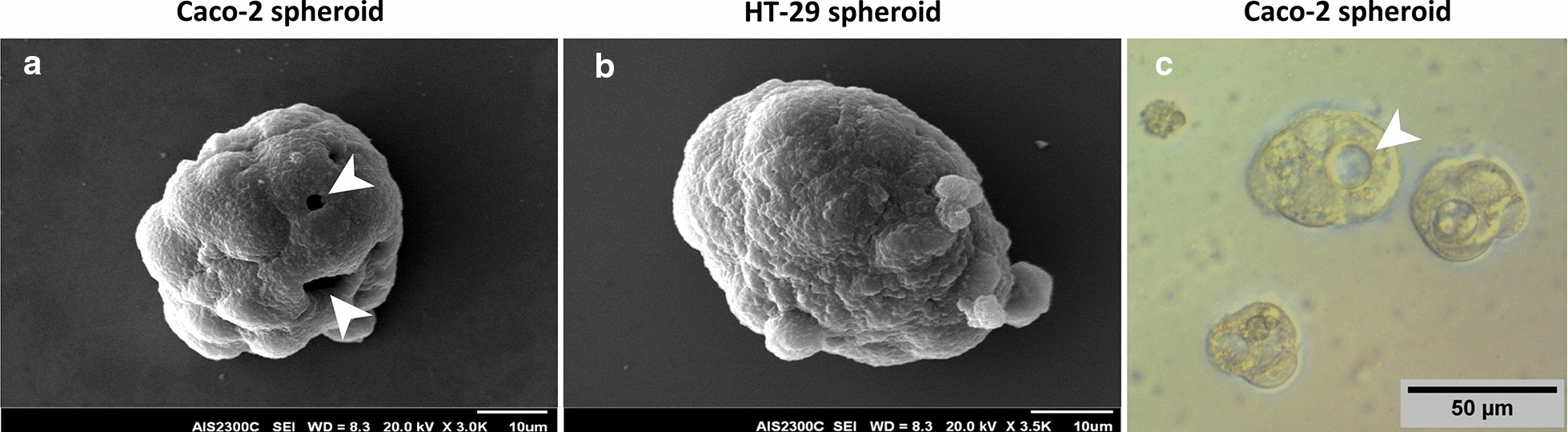
Scanning electron microscope (SEM) imaging of HT-29 and Caco-2 derived spheroids. SEM imaging was performed to assess spheroid morphological parameters in more details. Representative SEM images of spheroids shows roundness structure and smooth surfaces of both **a** Caco-2 and **b** HT-29 derived spheroids so that it is not possible to distinguish of individual cells. The hollow structures (arrows) in Caco-2 spheroids is its obvious difference compared to HT-29 spheroids with conglomerated appearance. **c** Representative phase contrast images of hollow core in Caco-2 derived spheroids. Arrows indicate hollow core of Caco-2 spheroids

In order to assess spheroid morphological parameters in more detail and increase the spatial resolution, micrographs of spheroids were acquired by SEM. Both HT-29 and Caco-2 colonospheres showed continuous and smooth surfaces without any plasma membrane projections or microvilli so that it was hard to distinguish individual cells (Fig. 3a, b). Interestingly, the hollow structures were observed in many of Caco-2 spheroids that were its obvious difference compared to HT-29 spheroids with conglomerated appearance (Fig. 3a–c).

### Enrichment of cancer stem cell-like sub-populations in generated spheroids

To investigate CSC enrichment in formed spheroids as compared to their adherent counterparts, we applied three separate techniques: (1) secondary sphere formation capacity whereby self-renewal properties of CSCs are exploited for maintaining stem-like features of cancer cells; (2) assessment of key stemness genes expression including *SOX2*, *C-MYC*, *OCT4*, *KLF4* and *NANOG*, as master regulators of pluripotency and self-renewal capacity in CSCs; and (3) quantifying the expression of potential CSCs surface markers by flow cytometry.

**Fig. 4:**
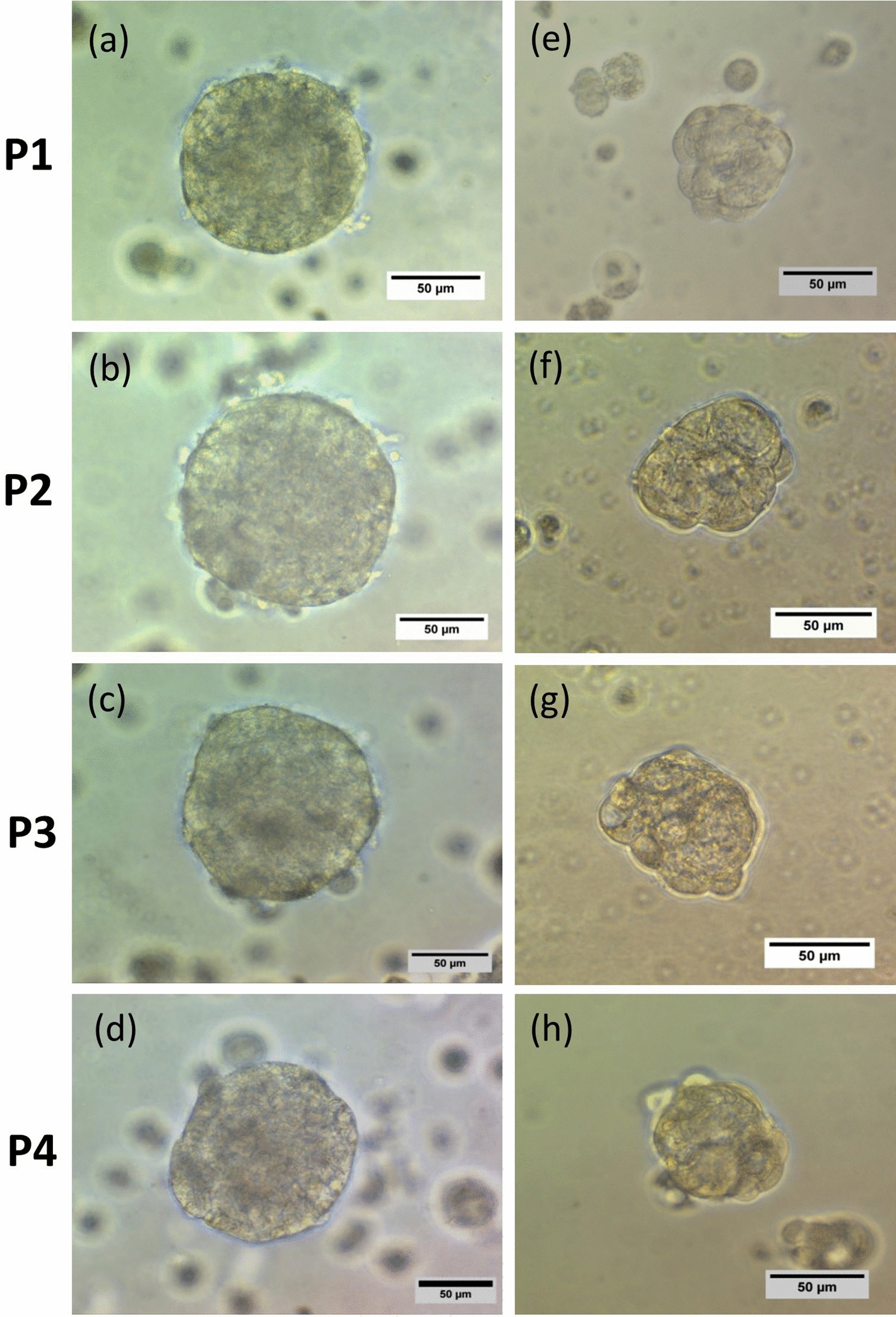
Serial spheroid formation capacity of colonospheres. To evaluate the self-renewal properties of generated spheroids, secondary spheroid formation was performed by serial passaging. Cells derived from **a–f** HT-29 and **e–h** Caco-2 spheroids generated spheroids for four consequence passage (P1–P4). There were no obvious differences in size and morphology of spheroids in different passages and their derived cells maintain their self-renewal capacity over several passages

**Fig. 5:**
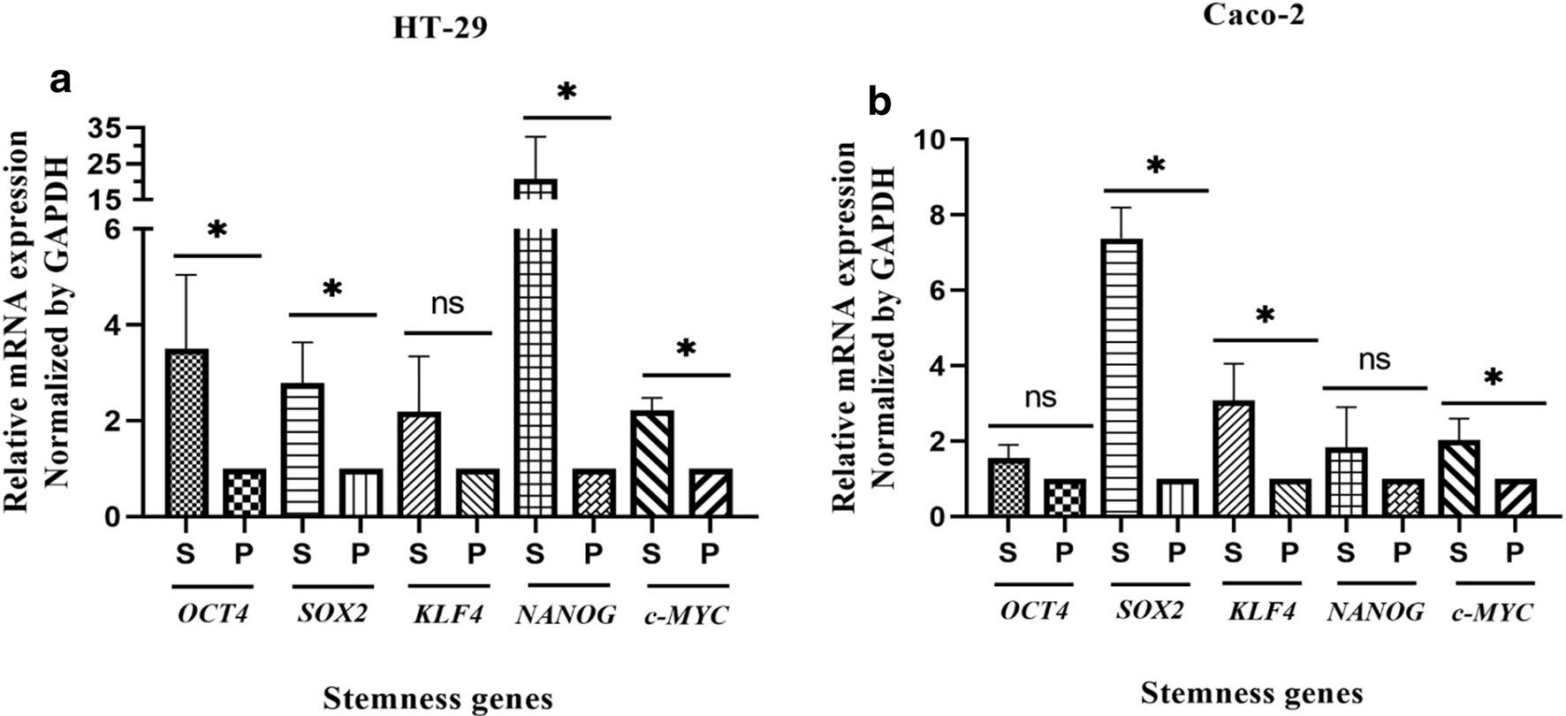
Increased expression of stemness regulator genes in HT-29 and Caco-2-derived spheroids compared to adherent counterparts. **a** Quantitative real-time PCR analysis of HT-29 spheroids revealed the high expression of key stemness genes *OCT4*, *SOX2*, *NANOG* and *C-MYC* compared to HT-29 parental cells with highest expression level of *NANOG* (*p-value* < *0.03*)*.* **b** Caco-2 spheroids showed an increased expression of *SOX2*, *C-MYC* and *KLF4* compared to Caco-2 parental cells with highest expression of *SOX2* (*p-value* < *0.029*). Relative gene expression was evaluated following 10 and 4 days of culture for HT-29 and Caco-2 spheroids, respectively. Data are presented as mean ± SD from four independent experiments as * = P < 0.05, ns = not significant

A secondary sphere formation ability of generated spheroids was investigated by serial passaging. Seeding of single cells from early passage of spheroids showed that spheroids derived from both HT-29 and Caco-2 cells still had the capacity to generate spheroids, even after four sub-cultures (Fig. 4a–h). Thus, these data suggest that spheroids maintain their self-renewal capacity over several passages. Furthermore, RT-qPCR analysis confirmed a definitive upregulation in most examined stem cell-related genes in both HT-29 and Caco-2 spheroids as compared to the 2D monolayers (Fig. 5a, b). HT-29 spheroids displayed the significant upregulation of *SOX2*, *C-MYC, NANOG* and *OCT4* (*p-value* < *0.03*) stemness genes compared to HT-29 parental cells with highest expression level of *NANOG* (*p-value* < *0.03*) (Fig. 5a). Whereas, Caco-2 spheroids showed significant high expression of *SOX2*, *C-MYC* and *KLF4* compared to Caco-2 parental cells with highest expression of *SOX2* (*p-value* < *0.029*) (Fig. 5b)*.*

**Fig. 6:**
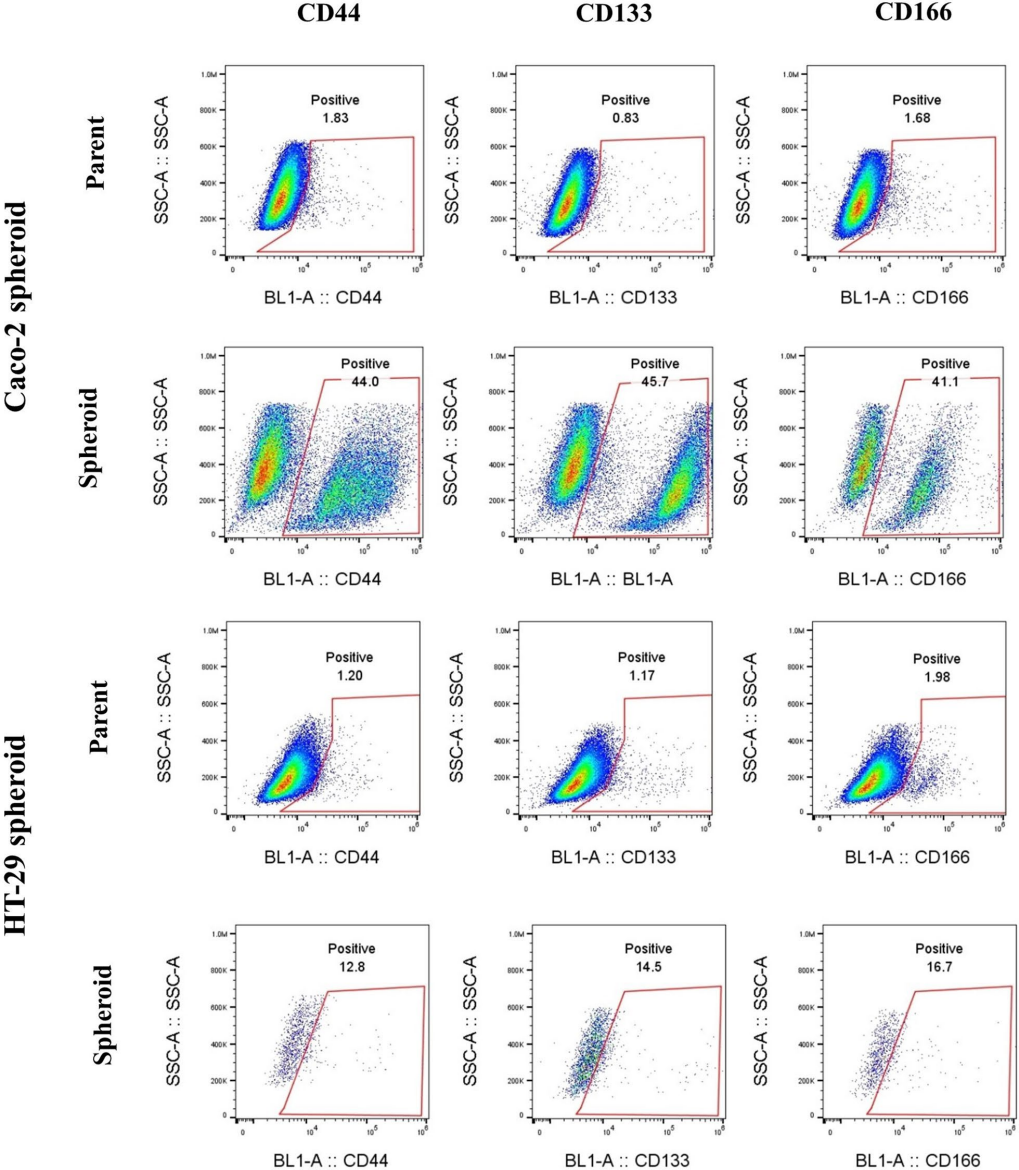
Flow cytometry analysis of CRC-CSC markers expression in HT-29 and Caco-2 spheroids compared to their parental cells. Representative flow cytometry plots confirmed both HT-29 and Caco-2 spheroids show higher expression of CD166, CD44 and CD133 CRC-CSC markers as compared to their parental cells. Upregulation of CSC markers in spheroids as compared to their differentiated counterparts was predominant in Caco-2 spheroids than HT-29 spheroids. Dot plots show the expression of each marker in one representative experiment

To further corroborate these findings, the expression of putative CSC surface markers CD166, CD44 and CD133 were investigated. Similar to our observations from stemness genes expression analysis, flow cytometry analysis also revealed that the expression of CD166, CD44, CD133 CSC markers were elevated in both spheroid models compared to differentiated 2D cultures which was statistically significant (CD166, CD44, CD133 (*p-value* < *0.03*) in HT-29 spheroids and CD166 (*p-value* < *0.0008*), CD44 (*p-value* < *0.0004*) and CD133 (*p-value* < *0.03*) in Caco-2 spheroids) (Fig. 6a, b) (Table 2).

**Table 2:** The expression percentage of CD44, CD166, and CD133 common CRC-CSCs markers in spheroids compared to their parental cells

Markers	Cell types			
	HT-29		Caco-2	
	Parental	Spheroid	Parental	Spheroid
CD44%	1.80 ± 0.77	19.3 ± 6.60^*^	1.59 ± 0.28	41.05 ± 3.53^***^
CD133%	1.35 ± 0.19	11.75 ± 4.69^*^	1.25 ± 0.45	28.25 ± 17.45^*^
CD166%	1.66 ± 0.37	22.40 ± 5.92^*^	1.78 ± 0.18	37.15 ± 4.02^***^

Data are shown as mean ± SD of four independent experiments. A significant upregulation of CSC markers in spheroids compared to parental cells are presented as * = P < 0.05, *** = P < 0.001, ns = not significant

### CSC-like enriched spheroids exhibited high expression of multi-drug resistant (MDR) genes

**Fig. 7:**
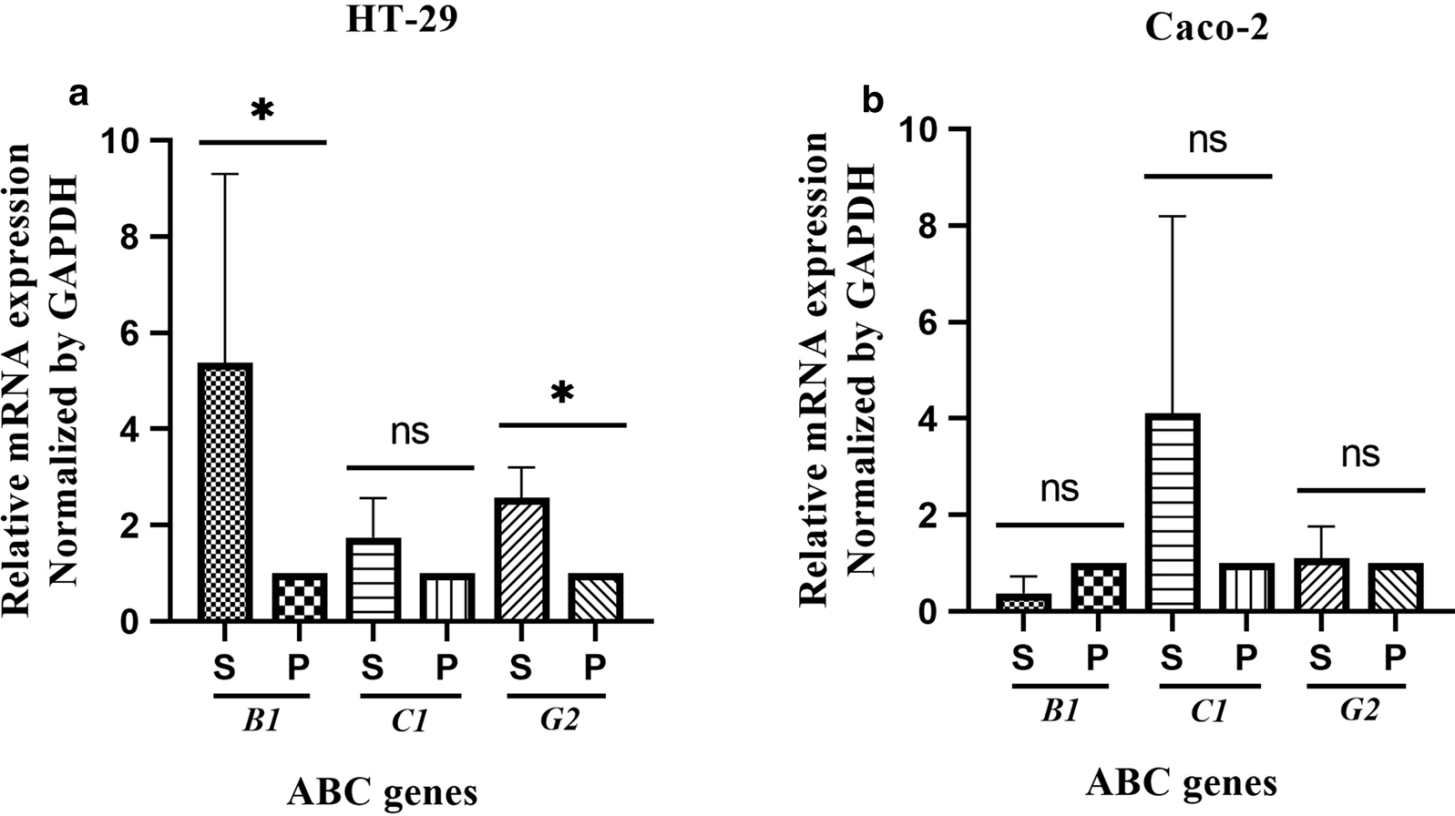
The expression profile of drug resistance genes in HT-29 and Caco-2 derived spheroids compared to their adherent counterparts. The comparative real-time PCR analysis of ABC transporter genes; *ABCB1*, *ABCC1* and *ABCG2* in generated spheroids and their parental cells showed **a** the higher expression of *ABCB1* and *ABCG2* in HT-29 derived spheroids than HT-29 parental cells, the expression of *ABCC1* was also increased, although this increase was not significant (*p*-*value* = 0.14), **b** whereas there was not observed significant differences between Caco-2 spheroids and their parental cells. Data are presented as mean ± SD from four independent experiments as * = P < 0.05, ns = not significant

Resistance against chemotherapeutics is another key feature of CSCs that is mediated by a family of ABC proteins. Accordingly, we assessed the expression pattern of a panel of major multi-drug resistant (MDR) genes; *ABCB1*, *ABCC1* and *ABCG2* in generated spheroids compared to parental cells. Specifically, HT-29-derived spheroids exhibited a significant higher expression of both *ABCB1* and *ABCG2* compared to HT-29 parental cells (*p-value* = *0.02*). The expression of *ABCC1* was also increased in HT-29 derived spheroids than their parental cells, although this increase was not significant (*p-value* = *0.14*) (Fig. 7a). In spite of the ABC expression pattern of HT-29 spheroids, Caco-2 spheroids did not show any significant differences in expression patterns of *ABCB1*, *ABCC1* and *ABCG2* compared to Caco-2 parental cells (*p-value* > *0.99*) (Fig. 7b).

### CRC spheroids were driven toward the EMT compared to 2D adherent cells

**Fig. 8:**
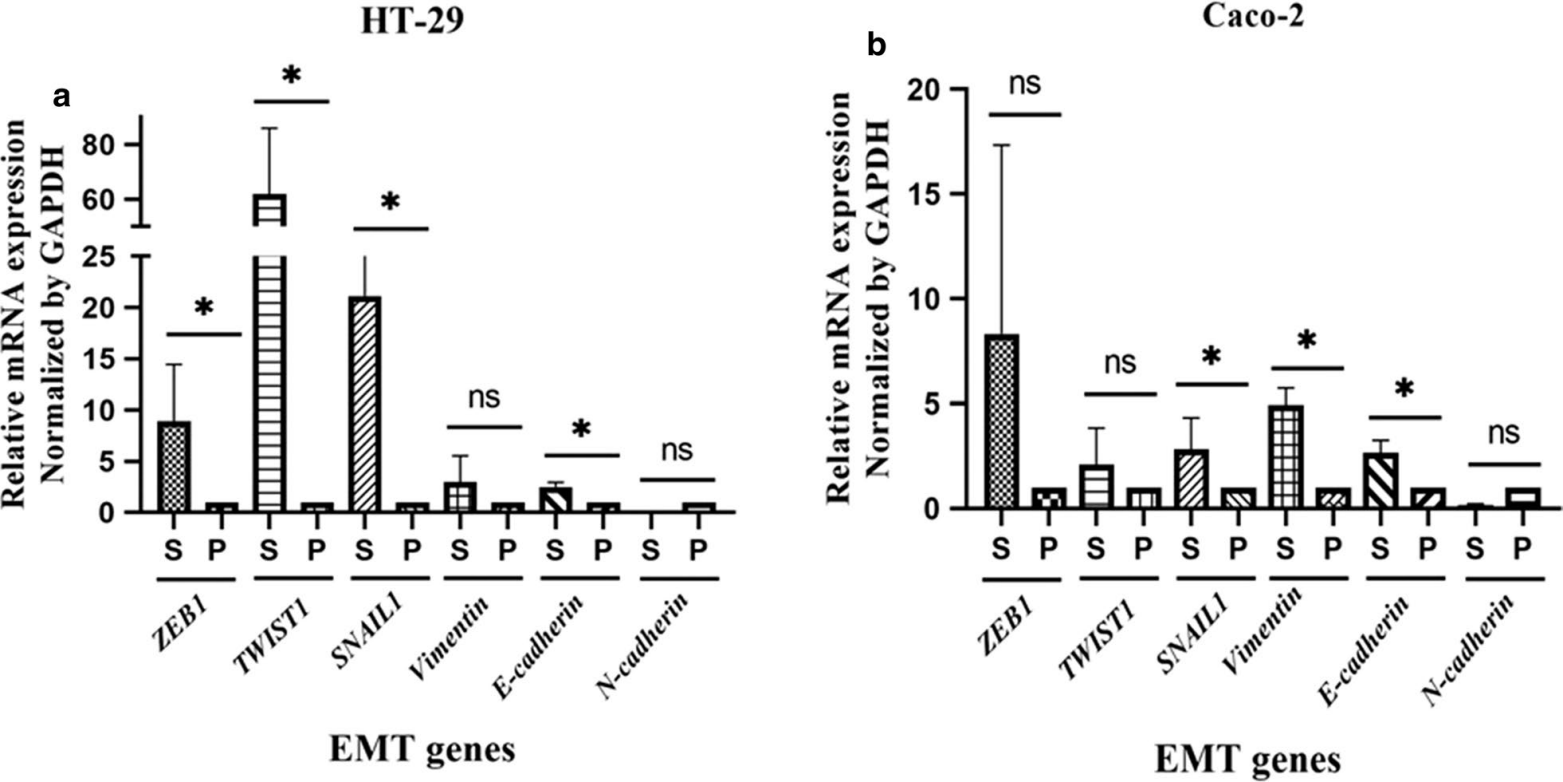
The expression profile of EMT regulator genes in HT-29 and Caco-2 derived spheroids compared to adherent counterparts. Graphs showing relative expression of genes involved in EMT; *Vimentin*, *SNAIL1*,*TWIST1*, *N-cadherin*, *E-cadherin* and *ZEB1* in generated spheroids. **a** The expression of *ZEB1*, *TWIST1*, *SNAIL1* and *E-cadherin* was significantly higher in HT-29 spheroids than parental cells. **b** Caco-2 spheroids showed higher expression of *SNAIL1*, *Vimentin* and *E-cadherin* compared to parental cells. Data are presented as *P < 0.05, ns = not significant

To characterize the EMT properties in generated spheroids, we next compared the expression profile of EMT inducer genes including *TWIST1*, *SNAIL1*, *ZEB1*, *Vimentin*, *E-cadherin* and *N-cadherin* in both spheroids and parental cells. The expression of EMT-promoting genes; *ZEB1*, *TWIST1* and *SNAIL1* were significantly up-regulated in HT-29 spheroids compared to 2D monolayer cultures (*p-value* = *0.03*). Unexpectedly, the expression of *E-cadherin* was up-regulated in HT-29 spheroids (Fig. 8a). A significant up-regulation of *SNAIL1* and *Vimentin* were also observed in Caco-2 spheroids than their parental cells (*p-value* < *0.05*). Although *ZEB1* showed a higher expression in Caco-2 spheres than 2D counterparts, this upregulation was not statistically significant (*p-value* > *0.05*) (Fig. 8b). On the contrary, the expression of *N-cadherin* was down-regulated in both HT-29 and Caco-2 spheroids compared to parental cells (Fig. 8).

### Discussion

To overcome the treatment failure due to the cancer recurrence and metastasis related to CSCs biological features and their interference with intrinsic drug resistance mechanisms, design of appropriate preclinical models is one of the most pressing issues to test targeted therapies [35, 36]. Identification of the ideal tools to clarify CSC biology is still proceeding, and great efforts have been paid to improve the isolation and treatment modalities in CSCs research [37]. Spheres culture in which CSCs are trapped and enriched, has been recommended as an extremely effectual CSC isolation method for cancer cell lines and solid tumors [37–39]. Such an anchorage independent sphere forming system under non-adherent, nutritional deficiency and serum free conditions prevents the differentiation of stem-like cells and leads to eliminate non-CSCs cells through apoptosis [40–42]. The superiority of this method is based entirely on the intrinsic properties of CSCs and leads to enriching the CSC subpopulations regardless of the expression patterns of cell surface markers [43, 44]. Moreover, spheroid cultures offer an ideal platform for routine toxicity and drug efficacy testing to determine safe exposure doses in designed cellular models [45–48]. In addition, previous studies have shown that different cancer cell lines form spheres with distinctive morphological and functional features based on sphere-formation method [49–51]. Hence, the status of morphological characteristics, CSC properties and genes expression profile of EMT and drug resistance is important in selecting appropriate model.

In this regard, in the current study, the forced floating using low attachment plates and hanging drop methods, as more applicable and feasible methods were used to generate CSC-enriched spheroids from Caco-2 and HT-29 cell lines, respectively. We chose these two cell lines because of their widespread use in research and drug discovery. The Caco-2 cell line is widely used in pre-clinical investigations, drug permeability and solubility studies and nanoparticle translocation. The cell line is accepted by the U.S. Food and Drug Administration (FDA) to support new drug applications. In addition both Caco-2 and HT-29 cells are widely applied in the studies of intestinal transport and prediction of bioavailability [52–54].

To generate spheroids, serum-free condition was applied to prevent the differentiation of stem-like cells and elimination of non-CSCs population using suspension culturing in low attachment plates [55–58]. Although both cell lines showed capacity to form spheroids, the HT-29 cells generated loose and disintegrating spheres in poly-HEMA plates, whereas, they formed compact spheroids when they were cultured as hanging drops. The comparison of morphological and molecular characteristics of Caco-2 and HT-29 derived spheroids has been summarized in Table 3.

**Table 3:** The comparison of morphological and molecular characteristics of Caco-2 and HT-29-derived spheroids

Characteristic	Cell type	
	Caco-2	HT-29
Sphere formation method	Free floating (suspension) culture	Hanging drop method
Complexity of setup	Simple (spontaneous formation)	Moderate
Cost and reproducibility	Low cost High reproducibility	Low cost High reproducibility Time consuming and extensive handling necessary
Spheroid formation rate (spheroidization time)	3 days	4 days
Spheroid structure maintenance	Moderate (stable for 5 days)	High (stable up to 12 days)
Morphological characteristics	Round-shape, hollow structures with bubble like structures	Round-shape, smooth surface and compact morphology
Secondary sphere formation ability	Yes	Yes
Spheroid size (average)	Medium (66.9 ± 14.88 µm)	Large (82.52 ± 22.56 µm)
CSC enrichment capacity	Yes	Yes
Up-regulated stemness genes	*SOX2, C-MYC, KLF4*	*SOX2, C-MYC, OCT4, NANOG*
CSCs surface markers up-regulation	+ +	+
MDR genes up-regulation	No	Yes (*ABCB1 and ABCG2*)
Up-regulated EMT genes	*SNAIL1, E-cadherin, Vimentin*	*TWIST1, SNAIL1, ZEB1, E-cadherin*

Generation of dense and practical spheroids strongly depend on cellular interactions through the gap junctions between single cells [59, 60]. Hence, hanging drop method was selected to generate HT-29 spheroids with compact structure due to its advantages in improvement of cells accumulation and adhesion potential with least damage to the spheres through gravitational force. Our observation was in line with the previous study in rat pancreatic beta cells which hanging drop method enhanced connexin protein accumulation and generation of tight spheres [60]. Moreover, murine and human brain tumor cell lines showed more consistent structures in brain tumor spheroids with applying of hanging drop method compared to spinner culture [61]. Therefore, it seems that different cell lines can be compatible with different spheroid formation methods, as in our study as well; hanging drop and low attachment poly-HEMA coated plates were suitable, rapid and time efficient for spheroid formation from HT-29 and Caco-2 cells, respectively. Some limitations of hanging drop including possible shattering of spheroids due to mechanical shock in transferring of spheroids to the conventional culture plates and high spheroid-diameter variation compared to low attachment culture can lead to changes in initiating characteristics in HT-29 spheroids. Whereas, forced floating method using low attachment culture with more reproducibility than hanging drop could be the most suitable method for high-throughput screening. Therefore, appropriate cells and spheroid formation methods must be accurately chosen based on aim of study and tumor type.

Although both spheroids derived from HT-29 and Caco-2 cells exhibited similar roundness and smooth surfaces, the light and electron microscopic examination revealed some differences. The Caco-2 spheroids presented hollow core structure as a distinct morphological feature which has been reported before by others [62, 63]. Samy et al. [64], also found that after 5 days culturing in Matrigel, the Caco-2 cells self-organized into intestinal epithelial-like cells as spheroids with a confluent monolayer surrounding a hollow lumen. This hollow structure in Caco-2 cells is account to the functional polarization through E-cadherin-dependent cell–cell adhesion [63, 65]. Since the hollow core is the common feature of the Caco-2 spheres formed by different methods [62, 63], it can infer that this feature is cell line dependent and may not be related to the sphere formation methods.

The Caco-2 spheroids features including the expression of drug transporters similar to human intestinal and also their ability to recreate the spatial organization similar to intestinal epithelial cells have been made them as an improved platform in drug screening [64, 66, 67]. However, Caco-2 spheroids with round shape and hollow lumenized morphology have widely employed as a reproducible in vitro model for studying intestinal features and functions as well as intestinal drug metabolism and uptake [64, 68], they are not compatible with anti-cancer drug researches which are based on evaluation of the spheroid size reduction and drug penetration into inner layers. The roundness and smooth surface of spheroids which hide individual cells, is ascribed to high extracellular microenvironment (ECM) secretion and strong cell–cell adhesion, and could help analysis of drugs efficacy more accurately [49, 69]. Accordingly, HT-29 spheroids with compact round shape may be more suitable platforms for anti-cancer drug testing in CRC than Caco-2 spheres. In general, in order to create more practical and functional CRC spheroid models, it is essential to characterize the gene expression alterations, invasion, and drug transporters in spheroids to standardize them based on research requirements [61, 64, 70–72]. Hence, CRC spheroid models were further assessed to determine their potential in enrichment of CSCs-related characteristics, including the cell surface marker patterns, serial sphere formation capability, and gene expression profiles of multipotency, EMT and drug resistance transporters compared to the parental cells. Our findings showed that the expression levels of stemness genes could be affected by CRC spheroid culture, and both spheroids displayed similar high expression levels of pluripotent stem cell genes (*KLF4*, *OCT4* and *C-MYC*) when compared to their parental counterparts, while drastic higher expression level of *SOX-2* and *NANOG* was found in Caco-2 and HT-29 spheres, respectively. It has been also documented that the overexpression of *NANOG* as an oncogene along with *OCT4* is a prominent characteristic of CSCs and is associated with EMT transition of CSCs and drives tumor progression and poor prognosis in patients with breast and colorectal cancer [73–78]. Furthermore, many studies have proved that *SOX-2* overexpression is correlated with self-renewal capacity, a poorly differentiated-aggressive phenotype and clinicopathological characteristics of CRC patients [79–81]. Therefore, high expression levels of stemness genes, specially, *SOX2* and *NANOG* that were observed in our CRC spheroids could be served as an indicator of self-renewal potential.

We extended our study by assessing the expression of CSC surface markers in CRC spheroids compared to the parental cells. Our findings independently corroborated the expression profiles of stem cell-related genes which evidenced by enriching the CD166, CD44, CD133 positive populations of CSCs in CRC spheroids. This was in line with other reports in various cancers, where non-adherent spheroid cultures possessed more characteristics of CSCs [44, 82–85]. CD44 as a transmembrane adhesion receptor for hyaluronic acid, binds to the ECM and plays an important role in matrix adhesion in response to a cellular microenvironment and is implicated in enhancing cellular aggregation [86, 87]. CD166 and CD133 are also cell adhesion molecules which along with CD44 increase the clonal formation capacity in CRC and are related to CSC properties [85, 88]. The cell adhesion proteins are participate in directing cell polarity and asymmetric stem cell division in development [89]. Although, there are limited studies on Caco-2 spheroids, we conclude that the higher expression of CD44, CD133 and CD166 as robust CSC markers in cells derived from Caco-2 spheroid might be related to specific cellular junctions in these spheroids, which provide signals via molecular crosstalk between these cells for maintenance of CSC phenotype in Caco-2 spheroids. In addition, due to the absence of CSCs in necrotic core of spheroids [90], we assume that many of cells in HT-29 spheroids that were negative for expression of CSC markers may belong to this layer, whereas, in case of Caco-2 spheroids, there were hollow core instead of necrotic layer. Hence, this feature might account for predominant expression of CD44, CD166 and CD133 CSC markers observed in Caco-2 spheroid-derived cells as compared to cells derived from HT-29 spheroid.

Moreover, serial sphere formation which has been applied as an appropriate platform for long-term expansion of cells with self-renewal capacity and imitates the tumor heterogeneity [24], was maintained in CRC spheroids cells during the long-term cultures. Thus, these results further advocated the efficiency of the serum free and non-adherent condition in enrichment of CSCs. The complexity of cross-talks between stem cell–related genes and EMT are still unclear and need to be more clarified. To determine the association of the EMT genes expression level with the enriched CSC nature in spheroids, we compared the expression of *TWIST1*, *SNAIL1*, *ZEB1*, *Vimentin*, *E-cadherin* and *N-cadherin* in spheroids than parental cells. Our results showed that in spite of up-regulation of *TWIST1*, *SNAIL1*, *ZEB1* and *Vimentin*, unexpectedly *E-cadherin* was up-regulated and *N-cadherin* was down-regulated in spheroids which are in contrast to the results of EMT process from other similar studies [91–94]. Several reports suggested the strong correlation of *E-cadherin* down-regulation and *N-cadherin* up-regulation as the main hallmark of EMT [76, 91–95]. It can be postulated that the expression level of EMT genes, might be varied widely due to the absence of definite correlation between the gene transcripts levels and their corresponding proteins and do not necessarily reflect their protein levels in CSCs [96–100]. In addition, Jolly, Jia et al. displayed that the partial EMT is associated with stemness [101]. Hence, deciphering the coupling of EMT and stemness needs to be further investigated.

The increased expression of ATP-binding cassette (ABC) transporter genes such as *ABCB1*, *ABCC1*, and *ABCG2*, as other CSC-related characteristic is involved in regulation of self-renewal and multidrug resistance in ovarian and colon cancer cell lines [102–107]. In agreement with Collura et al. [108], our results also demonstrated significant increase in the expression of *ABC B1* and *G2* genes in HT-29 spheres compared to monolayers and Caco-2 spheres which further verified the CSCs enrichment.

Although the results of our study present robust and reproducible spheroid models for CSC research, these findings should be considered in the light of some limitations, including evaluation of the spheroids of only two CRC cell lines and also performing additional functional analysis on metastatic capacity, ECM remodeling, chemoresistancy and dormancy. This information would provide new insight into the efficiency of the CRC spheroid model in CSC research.

## Conclusions

In summary, we present here the first study demonstrating CSC-enrichment in Caco-2 cells without using special scaffold or biochemical materials. Despite the hollow core structure and no increased expression of ABC transporter genes in Caco-2 spheroids, our findings indicated that spheroids culture from both HT-29 and Caco-2 cell lines is capable to enhance the expression of genes involved in CSCs regulation. It enables us to recreate the complexity of in vivo tumors including presence of CSCs subpopulation, resistance to chemotherapeutics and invasion potential and should be considered as more realistic CRC in vitro models for further investigation in CSC research.

## Data Availability

The analyzed data during the current study are available from the corresponding author on reasonable request.

## Abbreviations

2D: Two-dimensional
3D: Three-dimensional
ABC: ATP-binding cassette
ABCB1: ATP Binding Cassette Subfamily B Member 1
ABCC1: ATP Binding Cassette Subfamily C Member 1
ABCG2: ATP Binding Cassette Subfamily G Member 2
ALDH1: Aldehyde dehydrogenase 1
bFGF: Basic Fibroblast Growth Factor
C-MYC: C-MYC proto-oncogene
CRC: Colorectal Cancer
CSCs: Cancer Stem Cells
DMEM: Dulbecco's modified Eagle's medium
E-cadherin: Epithelial Cadherin
EGF: Epidermal Growth Factor
EMT: Epithelial-to-Mesenchymal Transition
FBS: Fetal bovine serum
FDA: Food and Drug Administration
GAPDH: Glyceraldehyde-3-phosphate dehydrogenase
KLF4: Kruppel-Like Factor 4
MDR: Major Multi-drug Resistant
NANOG: Nanog homeobox
N-cadherin: Neural Cadherin
OCT4: Octamer-binding transcription factor 4
PBS: Phosphate buffer saline
poly-HEMA: Poly-2-hydroxyethyl methacrylate
RT-qPCR: Real-Time Polymerase Chain Reaction
SEM: Scanning Electron Microscopy
SNAIL1: Snail family zinc finger 1
SOX2: SRY-Box Transcription Factor 2
TWIST1: Twist family bHLH transcription factor 1
ZEB1: Zinc finger E-box homeodomain 1
